# Detailed analysis of the male reproductive system in a potential bio-indicator species – The marine invertebrate *Galeolaria caespitosa* (Polychaeta: Serpulidae)

**DOI:** 10.1371/journal.pone.0174907

**Published:** 2017-04-03

**Authors:** Yonggang Lu, Robert John Aitken, Minjie Lin

**Affiliations:** Priority Research Centre for Reproductive Science, School of Environmental and Life Sciences, Faculty of Science, University of Newcastle, Callaghan, New South Wales, Australia; University of Hyderabad, INDIA

## Abstract

For the first time, this study has systemically investigated the male reproductive system in a sessile broadcast-spawning marine invertebrate, *Galeolaria caespitosa* (Polychaeta: Serpulidae), which has significant potential as a bio-indicator species of coastal marine pollution. The abdomen of *G*. *caespitosa* was divided by intersegmental septa into over 80 trunk segments. Each segment served as a germinal chamber with a C-shaped gonadal arrangement consisting of several distinct compartments: a seminiferous epithelium (SE) compartment located in the centre of the chamber, with each of its two ends connecting to a nurse cell (NC) compartment and then an efferent duct (ED) compartment. The SE compartment contained a multilayered seminiferous epithelium where spermatogenesis was initiated. Spermatids were released in pairs into the lumen of the SE compartment and then transported to the NC compartment where they underwent spermiogenesis with the support of secretory vesicles released by the nurse cells. Spermatozoa were stored in the ED compartment and subsequently released into the seawater through the vas deferens. Unlike vertebrates where germ cells differentiated in close proximity to the nurse cell population (i.e. Sertoli cells), the spermatogenic cells of *G*. *caespitosa* exhibited no direct contact with supporting cells at any spermatogenic stage. This finding suggested that the spermatogenesis in *G*. *caespitosa* was more dependent on intrinsic developmental programming than most species. Notwithstanding such differences, there were clear parallels between the male reproductive system of *G*. *caespitosa* and mammals, in terms of the structure and function. The independence of spermatogenic cells from supporting cells in *G*. *caespitosa* raised the possibility of inducing spermiogenesis *in vitro*, which would provide a useful tool to dissect the mechanisms underlying this complex cell differentiation process in invertebrates and other higher order animals.

## Introduction

*Galeolaria caespitosa* is an Australian native marine polychaete that ubiquitously inhabits along the south-eastern coast, with a continuous geographical distribution over 4,500 km [1]. Numerous characteristics that *G*. *caespitosa* possesses endow this species with the potential to serve as an excellent experimental model for toxicity testing and a bio-indicator species of coastal marine pollution. To be specific, this species can be easily collected from the intertidal region of the seashore, where marine organisms are subject to a comparatively intensive pressure derived from human activity. It is a sessile invertebrate accommodated within white calcareous tubes that form 5 to 15 cm thick assemblages on pier pilings and rock revetments with an extremely high density of up to 10 individuals per cm^3^ [2]. The sedentary lifestyle that *G*. *caespitosa* exhibits means that this species is chronically exposed to any hazardous substances in the local environment rather than the episodic exposure experienced by most vagile species [3, 4]. Its abundant occurrence allows this species to reflect the ecological status at the population level. *G*. *caespitosa* has a small body size, which is approximately 10 to 30 mm in length and 0.8 to 2.7 mm in thoracic width [5]. It is macroscopic, so microscopic equipment is not required if the study only involves whole-body analysis. This invertebrate is still much smaller than most marine vertebrates and, as a consequence, only low amounts of laboratory space and toxicants are required to implement toxicity testing. Such a small body size is also associated with an increase in the vulnerability of the host species to pollutants [6]. *G*. *caespitosa* is a filter-feeding species, which is able to accumulate hazardous substances through the uptake of suspended particulate matter during the water exchange involved in foraging activities [3, 7].

*G*. *caespitosa* is dioecious, gender being distinguishable by the orange colour of the female abdomen and creamy white colour of the male abdomen [8]. The conspicuous colours of their abdomens are due to the presence of viable free-floating gametes throughout the year [8, 9]. As a model broadcast-spawning species, *G*. *caespitosa* releases its gametes directly into the water column, where fertilisation occurs externally [8]. Under laboratory conditions, *G*. *caespitosa* can be readily stimulated to spawn gametes by simply extracting the worms from their tubes and exposing them to natural seawater in small peri dishes. Such a free-spawning strategy, coupled with the relative immobility of the parent organism, results in the gametes of *G*. *caespitosa* being directly exposed to pollutants in ambient water before, during and after insemination. The early embryogenesis in *G*. *caespitosa* follows a pattern of spiral cleavage and is extremely rapid, taking only 1.5 h for a fertilised oocyte to develop into a 2-cell embryo, 5 h into a blastula and 18 h into a trochophore larva [8, 10]. This feature enables researchers to access a virtually unlimited number of embryos with minimal incubation time and to perform rapid assessments to reveal the effects of marine pollutants on its early development. Indeed, previous ecotoxicological studies demonstrated that the gametes of *G*. *caespitosa* were vulnerable to trace metal exposure, reflected by dramatic declines in the success of fertilisation, embryogenesis and larval development [11, 12]. The rapid development and short life-cycle of *G*. *caespitosa* also allows this species to respond more promptly to any pollutant-induced sub-lethal effects on gene expression, fecundity, growth rate or longevity through changes in its population [13]. During life-cycle toxicity tests, more toxicants or effluent samples can be examined within a given time using this polychaete compared with alternative species with longer life-cycles. Moreover, *G*. *caespitosa* is amenable to laboratory holding and can be maintained for at least 2 weeks in aerated tanks without jeopardising the viability of its gametes. As a model species, it has an excellent ability to accommodate foreign environments as manifested by the high fertilisation rates (~ 98%) that can be achieved under laboratory conditions, without any acclimation period prior to testing [11, 14].

As spermatozoa are usually more vulnerable to toxic substances compared with fertilised oocytes or embryos, sperm bioassays are expected to be more effective than embryotoxicity tests, in terms of their capacity to detect low levels of marine contamination. For example, recently researchers demonstrated that the behaviours of spermatozoa in *G*. *caespitosa* could effectively reflect the subsequent success of fertilisation and could therefore be employed as a sensitive bio-indicator for marine pollution monitoring [15, 16]. Specifically, the study conducted by Schlegel et al. [16] indicated that sperm motility and swimming velocity significantly decreased when being exposed to both near-future (∆pH– 0.3) and far-future (∆pH– 0.5) ocean acidification scenarios; and the acid-treated spermatozoa further resulted in dramatically reduced rates of fertilisation. Such sensitive responses to minor changes in the pH of seawater demonstrated that the behaviours of spermatozoa in *G*. *caespitosa* could provide early warning alerts for ocean acidification. Further, Falkenberg et al. [15] established a bioassay system using the spermatozoa of *G*. *caespitosa* for water quality monitoring–Sperm Accumulated Against Surface (SAAS). This study revealed that motile spermatozoa of *G*. *caespitosa* could actively accumulate at the lower surface of tissue culture plates, while the immotile cells were unable to adhere to this structure. More importantly, a strong positive correlation was detected between the number of spermatozoa accumulated on the plate surface and the success of fertilisation, indicating that SAAS could reliably reflect the fertilising capacity of the spermatozoa [15].

To gain profound insights into the vulnerability of spermatozoa in *G*. *caespitosa* to pollutant-induced damage and facilitate future establishment of other biomonitoring approaches using sperm function and behaviour, a comprehensive knowledge of male reproductive biology in this species is indispensable. However, the structure of the male reproductive system, as well as the pattern of germ cell differentiation in *G*. *caespitosa* remains unknown at present. In the current study, a detailed analysis of the male reproductive system in *G*. *caespitosa* has been undertaken, revealing novel developmental features that support the use of this organism as a model species in an ecotoxicology context.

## Materials and methods

### Sample collection and maintenance

Aggregations of *Galeolaria caespitosa* were collected freshly at low tides between March and June from intertidal rock revetments at Merewether beach (32°56’34”S, 151°45’9”E), Merewether, New South Wales, Australia. The collection of this material was performed in accordance with NSW State legislature covering the collection of non-endangered and non- protected invertebrate species from the marine environment. The tubeworms were transported to the laboratory within 1 h of collection and maintained in an aerated polyethylene bucket with natural seawater obtained from the collection site. Samples were reared at constant room temperature of 20 ± 2°C and supplied with a 12-h light/12-h dark illumination cycle. All samples processed during this study were fixed within 24 h of collection.

### Serial paraffin sections for light microscopy

Male adults were carefully extracted from their calcareous tubes using fine forceps and fixed in 10% neutral buffered formalin (Sigma-Aldrich, Castle Hill, Australia) for at least 48 h at room temperature. Fixed worms were dehydrated through a graded series of ethanol solutions for 45 min each. To remove the dehydrating agent, the specimens were then washed in 50% xylene in acetone (v/v) for 45 min, followed by another two washes in 100% xylene. The specimens were infiltrated for four cycles and embedded in paraffin wax at the melting point of between 54–58°C. Serial sections were cut along the longitudinal body axis of animals at a thickness of 5 μm using a Leica RM 2145 microtome (Leica, Heidleberg, Germany) and mounted onto microscope slides. Every fourth serial section was double-stained with Harris’ haematoxylin and eosin. The slides were then viewed and photographed under a Zeiss Axiovert S100 inverted microscope with a Zeiss AxioCam MRm camera (Carl Zeiss, Oberkochen, Germany).

### Transmission electron microscopy

After being extracted from their calcareous tubes, whole worms were immediately immersed in 2.5% (v/v) glutaraldehyde (ProSciTech, Queensland, Australia) in artificial seawater (28.32 g NaCl, 0.77 g KCl, 5.41 g MgCl_2_.6H_2_O, 7.13 g MgSO_4_.7H_2_O, 1.18 g CaCl_2_ and 0.2 g NaHCO_3_ in 1 L distilled water) with an osmolality of 1,140 mOsm/kg and pH at 7.4 at room temperature for 1 h. The abdomen of each worm was cut into cubes no more than 1 mm^3^ in size. The specimens were then transferred to freshly made fixative and fixed at 4°C overnight. Fixed specimens were washed and post-fixed in 1% (v/v) osmium tetroxide in artificial seawater (pH 7.4) for 2 h. After dehydration through an ascending acetone series, the specimens were infiltrated with 50% (v/v) Spurr’s resin embedding medium (ProSciTech, Queensland, Australia) in absolute acetone on a rotator at room temperature overnight. The resin medium was then replaced with 100% Spurr’s resin and the specimens were infiltrated for a further 12 h. The specimens were then embedded in freshly made 100% Spurr’s resin in polyethylene embedding capsules or moulds (ProSciTech, Queensland, Australia) and polymerised in an oven at 65°C for 24 h. Resin blocks were cut into semi-thin sections on a Reichert Ultracut S Microtome (Leica, Wien, Austria) with freshly made glass knives or a diamond knife (Diatome, Bienne, Switzerland). Semi-thin sections were stained with 0.5% (w/v) toluidine blue (BDH Chemicals, Kilsyth, Australia) in absolute ethanol and observed under the Zeiss Axiovert S100 microscope (Carl Zeiss, Oberkochen, Germany). Ultra-thin sections at a thickness of 60 nm were cut and stained with aqueous saturated uranyl acetate and Reynolds’ lead citrate (BDH Chemicals, Poole, England) and examined under a JEM-2100EX II transmission electron microscope (JEOL, Tokyo, Japan) at 80 kV.

### Scanning electron microscopy

For SEM specimens, whole male worms were fixed in 2.5% glutaraldehyde and 1% osmium tetroxide, followed by dehydration through a graded series of ethanol solutions. In order to remove excess liquid in the specimens, critical point drying was employed using liquid carbon dioxide as the transition solvent. Dried specimens were mounted on anodised aluminium stubs, coated with gold-palladium using a SPI Module™ Sputter Coater and then examined under a Philips XL30 scanning electron microscope (Philips, Eindhoven, Netherlands).

### Live cell observation

Adult male *G*. *caespitosa* were extracted from their calcareous tubes and immersed in filtered natural seawater. The worms spontaneously released their spermatozoa on being exposed to seawater. They were allowed to sit for at least 10 min until the spawning ceased. After that, the worms were rinsed vigorously in seawater and transferred to clean seawater. More spermatozoa, along with a considerable amount of spermatids, were stimulated to release by puncturing the abdominal wall with a syringe needle and gently pressing the abdomen using a pair of forceps. The released sperm solution was collected using Pasteur pipettes and examined under the Zeiss Axiovert S100 microscope (Carl Zeiss, Oberkochen, Germany). Images of spermatids were captured using the equipped Zeiss AxioCam MRm camera (Carl Zeiss, Oberkochen, Germany).

### Statistical analysis

Cellular and nuclear diameters of spermatogonia and spermatocytes are presented as mean ± SE, calculated from at least 20 samples of each cell type. The statistical difference between the sizes of spermatogonia and spermatocytes was determined using the Student’s t-test. Differences were recognised as statistically significant if the *P* value was less than 0.05. All statistical analyses were performed using Microsoft Excel 2013 (Microsoft Corporation, Redmond, USA).

## Results

### Male reproductive system in *Galeolaria caespitosa*

**Fig 1:**
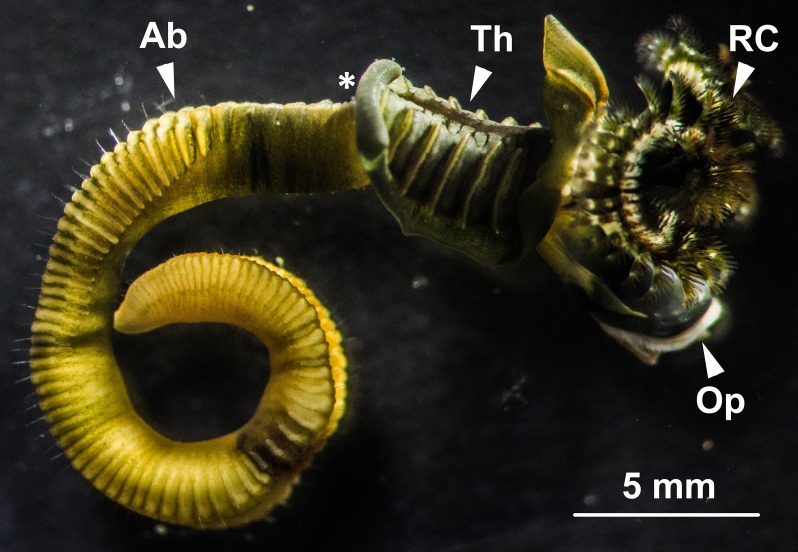
A male *G*. *caespitosa* extracted from its calcareous tube. *G*. *caespitosa* consisted of a conspicuous head bearing a radiolar crown (RC) and an operculum (Op), a thorax (Th) and a segmented abdomen (Ab). The main sperm duct (or vas deferens) lay along the mid-ventral region of the abdomen with an opening (*) located at the junction of the abdomen and thorax.

**Fig 2:**
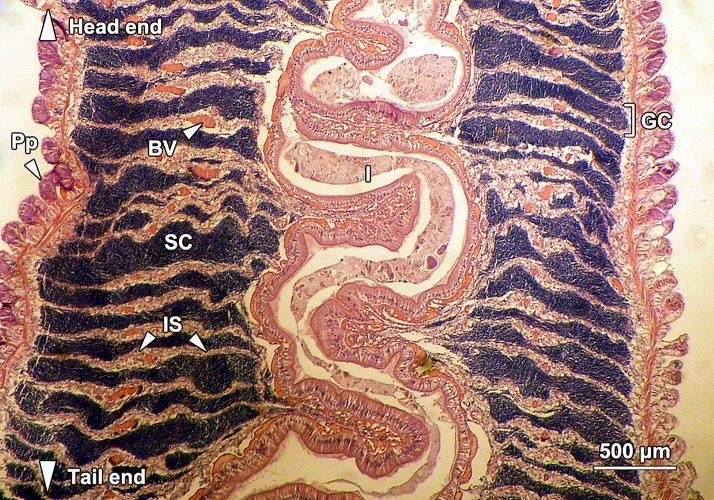
A longitudinal section through the abdomen of *G*. *caespitosa*. Internally, a coiled intestine (I) ran the whole length of the abdomen. The abdomen was divided into over 80 trunk segments by intersegmental septa (IS). Each segment externally bore a pair of parapodia (Pp). Ramified blood vessels (BV) were located within the intersegmental septa. The abdominal segments served as germinal chambers (GC) where the spermatogenesis was initiated. These germinal chambers were filled up with spermatogenic cells (SC) at various stages of spermatogenesis.

Male *G*. *caespitosa* was composed of a head bearing a prostomial radiolar crown (RC) and an operculum (Op), a six-segmented thorax (Th), and a creamy white abdomen (Ab; Fig 1). The abdomen of adult *G*. *caespitosa* was divided into over 80 trunk segments by intersegmental septa (IS) which consisted of a thin layer of interlacing connective tissue (Fig 2). Each abdominal segment surrounded an unsegmented intestine (I) and a coiled main artery that ran the whole length of the abdomen. Except for the first five to six segments next to the thorax, all other abdominal segments served as germinal chambers (GC) where spermatogenesis occurred. These chambers were constantly filled with sperm cells (SC) at various stages of spermatogenesis. The head-end intersegmental septum formed the “floor” of each germinal chamber, whereas the tail-end septum formed the “ceiling”.

**Fig 3:**
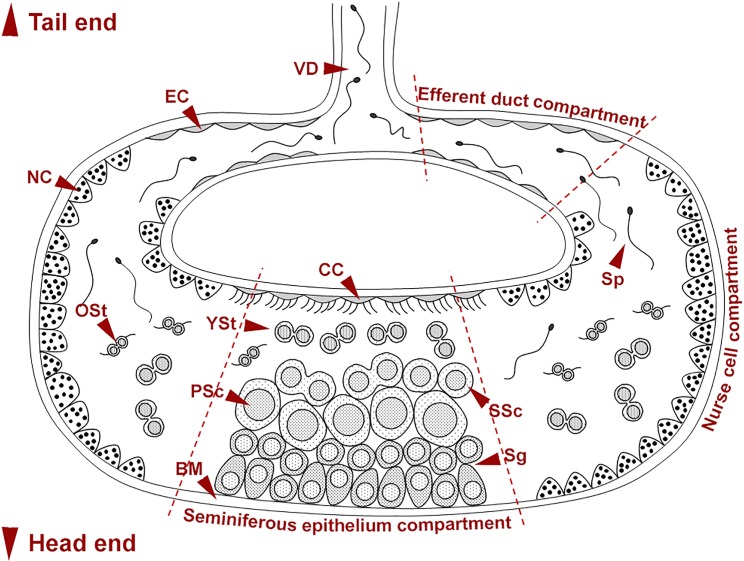
A schematic diagram showing a germinal chamber of the male reproductive system in *G*. *caespitosa*. The C-shaped germinal chamber was divided into three distinct compartments: one seminiferous epithelium compartment, two nurse cell compartments and two efferent duct compartments. Spermatogenesis occurred in the seminiferous epithelium compartment and spermatogonia (Sg) initially appeared on the basement membrane (BM) of the germinal chamber. The spermatogonia underwent a series of mitotic divisions and subsequently developed into primary spermatocytes (PSc), which further produced secondary spermatocytes (SSc) through the first meiotic division. Each secondary spermatocyte subsequently developed into a pair of young spermatids (YSt) through the second meiotic division. The paired young spermatids were released from the seminiferous epithelium and migrated into the nurse cell compartments where they underwent spermiogenesis and differentiated into older spermatids (OSt) and spermatozoa (Sp). The spermatozoa then migrated towards the efferent duct compartments and were finally released into the seawater through the vas deferens (VD). CC, ciliated cells; EC, squamous epithelial cells; NC, nurse cells.

Transversely, the germinal chambers appeared as a C-shaped tubule which consisted of three morphologically distinct compartments: one seminiferous epithelium (SE) compartment, two nurse cell (NC) compartments and two efferent duct (ED) compartments (Fig 3). The SE compartment was located at the centre of the C-shaped chamber connecting with the two NC compartments at each end. Then each of the NC compartments was linked to an ED compartment. Finally, the two ED compartments joined together to create an unsegmented vas deferens (VD), which ran through the whole abdomen with an opening situated at the joint between the thoracic and abdominal regions.

#### Seminiferous epithelium compartment

**Fig 4:**
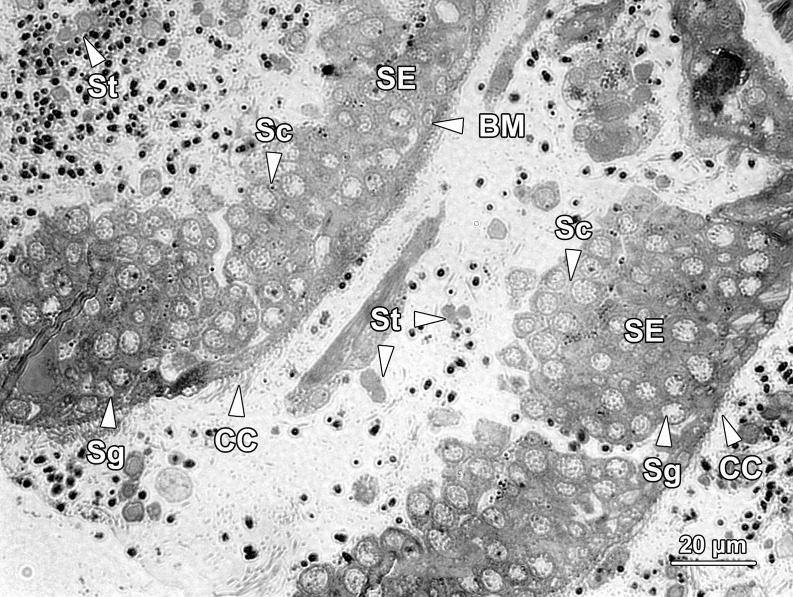
A longitudinal section of two neighbouring seminiferous epithelium compartments. The floor of the compartment was layered with seminiferous epithelium (SE), whereas the ceiling was covered by a monolayer of ciliated cells (CC). The seminiferous epithelium contained layers of spermatogonia (Sg) and spermatocytes (Sc). Spermatogonia occupied the initial layers near the basement membrane (BM), while spermatocytes with a larger size and paler colour were located in the upper layers close to the lumen. Paired spermatids (St) detached from the seminiferous epithelium and completed the remainder of spermatogenesis in the lumen of the germinal chamber.

**Fig 5:**
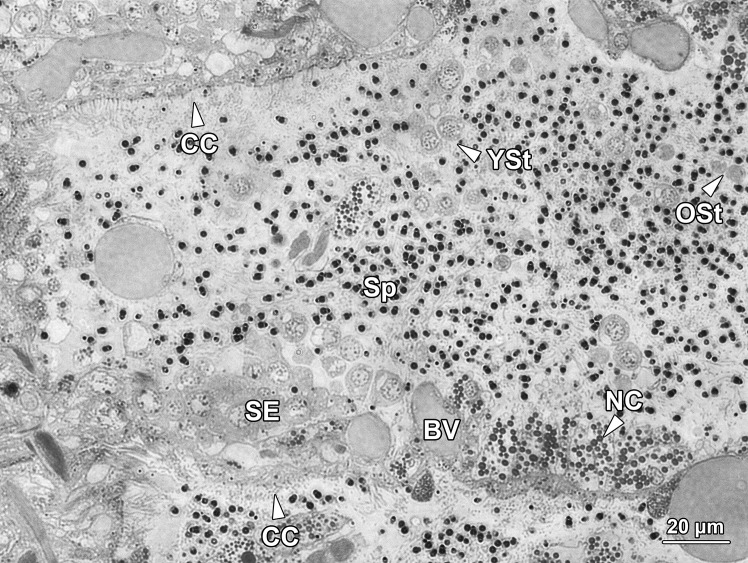
A longitudinal section of the connecting area between the seminiferous epithelium compartment (SE, on the left) and a nurse cell compartment (NC, on the right) in a germinal chamber. Young (YSt) and older spermatids (OSt) were observed floating freely in the lumen and mixed with numerous spermatozoa (Sp) and granules secreted by the nurse cells. Noticeably, neither nurse cells nor blood vessels (BV) exhibited any physical connection with germ cells.

Spermatogenesis was orchestrated in this compartment. The seminiferous epithelium (SE) sat on the basement membrane (BM) of the chamber floor and contained spermatogonia (Sg) and spermatocytes (Sc) only, which were densely packed in multilayers (Fig 4). Spermatogonia were located in the bottom layers, in close apposition to the basement membrane, while primary and secondary spermatocytes were in the top layers. Spermatids (St) were never found within the seminiferous epithelium, but were abundant in the lumen, close to the ceiling of the SE compartment (Fig 4). Thus, the mitotic and meiotic divisions of spermatogenesis occurred within the seminiferous epithelium, while the entire process of spermiogenesis, during which haploid round spermatids differentiated into functional spermatozoa (Sp), occurred in the lumen outside the epithelium. Blood vessels (BV) were frequently observed adjacent to the seminiferous epithelium, but there was no direct contact between them (Fig 5). No nurse cells were observed in the SE compartment. The ceiling of the SE compartment was covered by a monolayer of ciliated epithelial cells (CC; Figs 4 and 5), which facilitated the transportation of freely floating spermatids at various stages of development from the SE compartment to the NC compartments.

#### Nurse cell compartments

**Fig 6:**
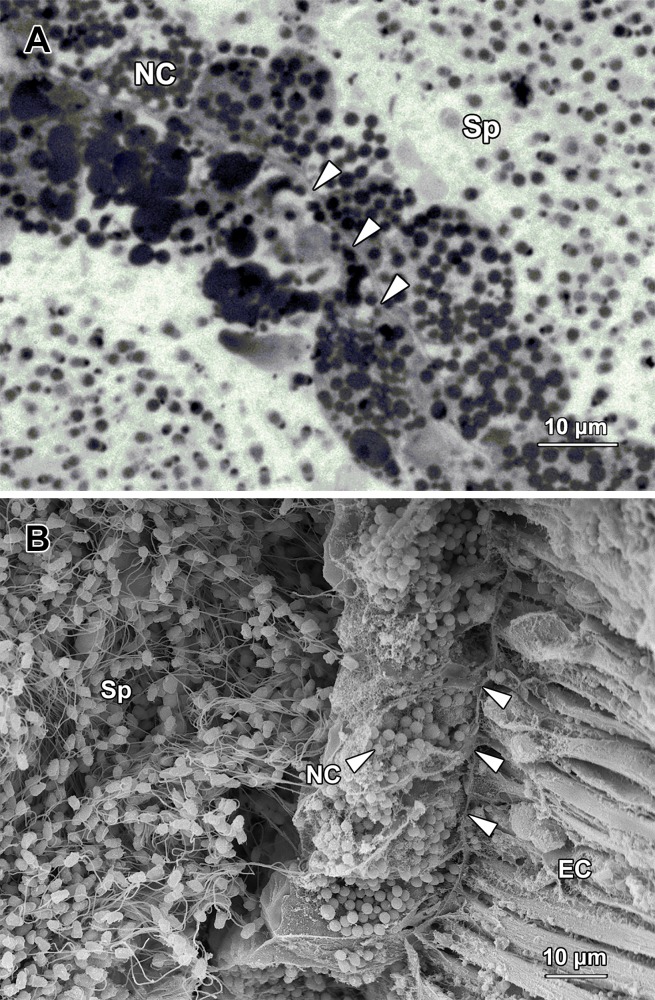
**(A) A light micrograph showing nurse cells** (NC) **lining the border** (arrowheads) **of two nurse cell compartments.** The nurse cells were filled with spherical granules which were released to the lumen to support the differentiation of spermatids. Sp, spermatozoa. **(B) A scanning electron micrograph showing a portion of a nurse cell compartment filled with spermatozoa** (Sp)**.** The nurse cells (NC) were densely distributed along the wall of the compartment (arrowheads), with epithelial cells (EC) of digestive tract on the other side.

The two NC compartments were located at each end of the SE compartment. The wall of this compartment was tightly packed with spherical- to ovoid-shaped nurse cells (Figs 5 and 6). These cells averaged 15.81 ± 0.70 μm in diameter and contained a large number of electron-dense spherical granules with a diameter of approximately 1.72 ± 0.055 μm (Fig 6). These nurse cells supported the differentiating spermatids by releasing their cellular contents into the lumen, rather than through any sort of direct contact. Noticeably, the lumen of NC compartments contained an enormous number of individual or paired spermatids at different stages of spermiogenesis, as well as spherical granules released from nurse cells (Fig 5).

#### Efferent duct compartments

**Fig 7:**
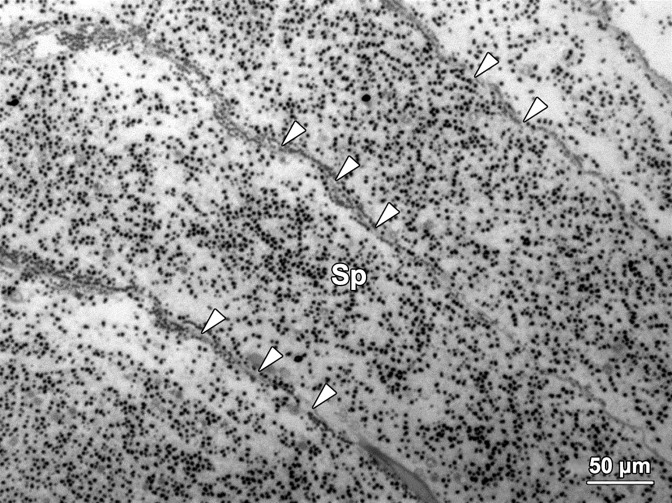
A light micrograph showing four neighbouring efferent duct compartments containing large numbers of spermatozoa (Sp) and spherical granules secreted by nurse cells. Each side of the intersegmental septa (arrowheads) in this compartment was covered by a thin layer of epithelial cells.

**Fig 8:**
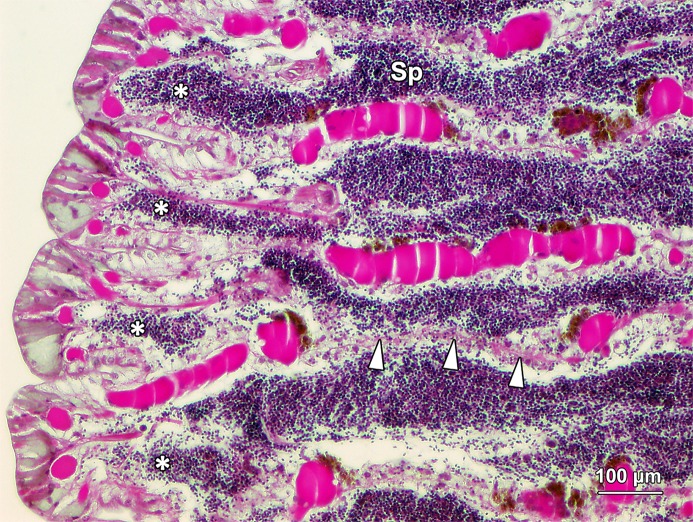
A light micrograph showing efferent duct compartments connecting to the vas deferens (*) at the terminus of each C-shaped germinal chamber. The wall of the efferent duct in this region (arrowheads) was comparatively thicker than other regions of this compartment. It consisted of muscle cells that facilitated migration of spermatozoa from the efferent ducts to the vas deferens.

The two ED compartments were located at each end of the C-shaped germinal chamber and connected the NC compartments with the vas deferens (Fig 3). The wall of ED compartments encompassed a layer of thin intersegmental septa and a layer of squamous epithelial cells (Fig 7). The lumen of ED compartments contained large numbers of spermatozoa (Sp) and few spermatids. The two ED compartments joined a thin, unsegmented vas deferens which lined the inner surface of the mid-ventral body wall. The wall of efferent duct at the junction of the ED compartment and vas deferens was thicker than that in other regions of this compartment (Fig 8). With the muscle contraction of the abdominal region, spermatozoa in each germinal chamber were transported from the efferent ducts into the vas deferens, and were finally released into the water column from the opening of the vas deferens situated at the joint between thoracic and abdominal regions (Fig 1).

### Germ cell differentiation in *G*. *caespitosa*

#### Pattern of spermatogenesis

**Fig 9:**
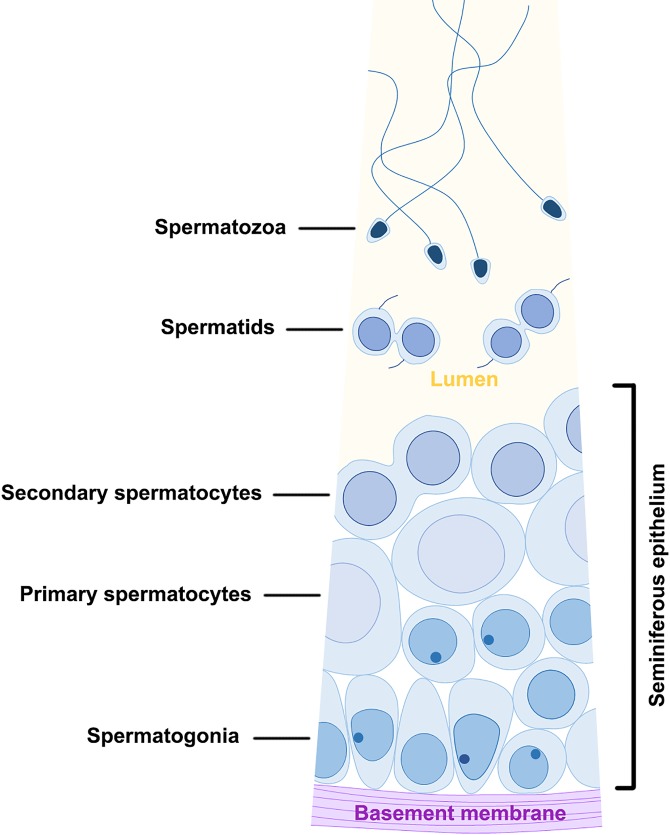
A diagram showing the pattern of spermatogenesis in *G*. *caespitosa*. Spermatogonia were in clusters and in close apposition to the basement membrane of the SE compartment. They underwent mitotic divisions and developed into primary spermatocytes while migrating away from the basement membrane. A primary spermatocyte divided into a pair of secondary spermatocytes through the first meiotic division and each secondary spermatocyte further divided into a pair of spermatids after the second meiotic division. The paired spermatids subsequently detached from the seminiferous epithelium and completed spermiogenesis while floating freely in the lumen of the germinal chamber. No direct contact between germ cells and nurse cells was observed at any spermatogenic stage.

Figs 3 and 9 collectively illustrated the pattern of germ cell differentiation in *G*. *caespitosa*. Spermatogenesis initially occurred in the seminiferous epithelium located on the floor of the SE compartment. Spermatogonia were basally located in the seminiferous epithelium but after a series of mitotic divisions, they ascended to the upper layers and divided into primary spermatocytes, which subsequently formed secondary spermatocytes through the first meiotic division. During the second meiotic division, each secondary spermatocyte divided into a pair of spermatids connected by a cytoplasmic bridge. These newly formed spermatids were maintained in pairs when they detached from the seminiferous epithelium and were released into the lumen of SE compartment. These paired spermatids completed the remainder of spermatogenesis while floating freely in the lumen of germinal chamber.

With the assistance of ciliated cells on the ceiling of each SE compartment, paired young spermatids (YSt; Fig 5) migrated into the NC compartments situated at both ends of the SE compartment. They were mixed with granules secreted by the nurse cells and continued to develop into older spermatids (OSt) and spermatozoa in the NC compartments. The newly formed spermatozoa (Sp) migrated towards and were stored in the ED compartments (Fig 7). After the completion of spermiogenesis, spermatozoa entered into the vas deferens with the help of the epithelial cells (EC) on the wall of ED compartments (Fig 3). Eventually, spermatozoa were released into seawater for fertilisation through the opening of vas deferens (VD) by rhythmic contractions of the vas deferens and abdominal muscles (Fig 1).

On the basis of both light and electron microscopic observations, the germ cells at various spermatogenic stages in *G*. *caespitosa* were classified as follows.

#### Spermatogonia

Spermatogonia could only be found in the bottom layers of the seminiferous epithelium located in the SE compartment of each germinal chamber. Based on their positions, cell size and ultrastructural morphology, spermatogonia were subdivided into two types.

**Fig 10:**
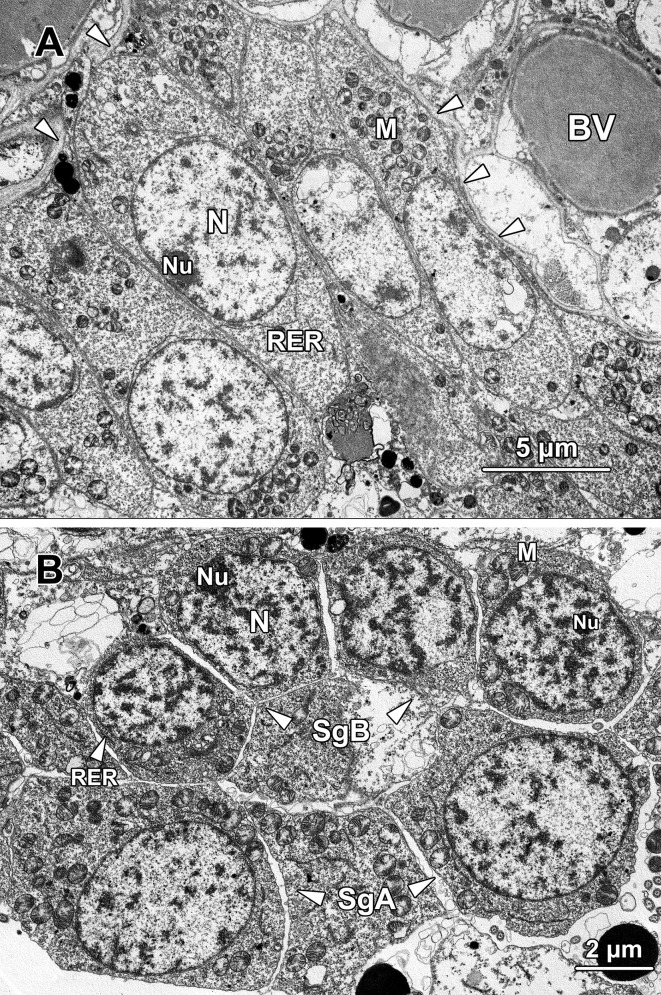
**(A) A transmission electron micrograph showing a layer of type A spermatogonia packed along the basement membrane** (arrowheads) **of the SE compartment.** The type A spermatogonia had no physical contact with the blood vessel (BV). Numerous mitochondria (M) tended to cluster in the basal cytoplasm while the nucleus (N) contained irregularly scattered clumps of chromatin blocks and an eccentrically located nucleolus (Nu). RER, rough endoplasmic reticulum. **(B) A transmission electron micrograph showing two type A spermatogonia** (SgA, the lower row) **and four type B spermatogonia** (SgB, the upper row)**.** Type B spermatogonia were usually found one or two layers away from the basement membrane. Irregularly accumulated chromatin could be observed lining the inner nuclear membrane and in the centre of the nucleus. A conspicuous nucleolus (Nu) was frequently be observed in the nucleus adhering to the nuclear membrane of type B spermatogonia. M, mitochondria; RER, rough endoplasmic reticulum.

Type A spermatogonia were firmly adherent to the basement membrane (Fig 10A). They were approximately 8.46 ± 1.63 μm in diameter with a nucleus about 5.2 ± 0.8 μm in diameter. These spermatogonia were ovoid to fusiform in shape, with an oval nucleus (N) filled up with mostly sparse granular euchromatin and a few randomly distributed heterochromatin blocks. One or two small nucleoli (Nu) could sometimes be observed in the nucleus. The cytoplasm of type A spermatogonia was abundant in volume, however, few organelles were observed except for a large quantity of oval- to round-shaped mitochondria (M) and a continuous profile of rough endoplasmic reticulum (RER) lying in close proximity to the nucleus. The mitochondria were preferentially congregated in the basal cytoplasm of type A spermatogonia which was the aspect directed towards the basement membrane.

Type B spermatogonia (SgB) were situated away from the basement membrane (Fig 10B); they were smaller in size but outnumbered type A spermatogonia (SgA) in the seminiferous epithelium, indicating they were the offspring of type A spermatogonia. Type B spermatogonia averaged 6.89 ± 1.04 μm in diameter and possessed a nucleus about 4.4 ± 0.69 μm in diameter. The differences in cellular and nuclear sizes between these two types of spermatogonia were statistically significant (P < 0.001). Morphologically, type B spermatogonia possessed relatively more ribosomes in the cytoplasm and the inner membrane of its nuclear envelope was scattered with more abundant heterochromatin blocks, which made the nuclear membrane less obvious compared with type A spermatogonia. Rather than being ovoid or fusiform in shape, the type B spermatogonia appeared more spherical. Similarly, they also contained a considerable number of mitochondria (M) in their cytoplasm and a conspicuous profile of rough endoplasmic reticulum (RER) surrounding the nucleus (Fig 10B). The nucleus (N) of type B spermatogonia was spherical in shape and contained a prominent eccentric nucleolus (Nu).

#### Spermatocytes

**Fig 11:**
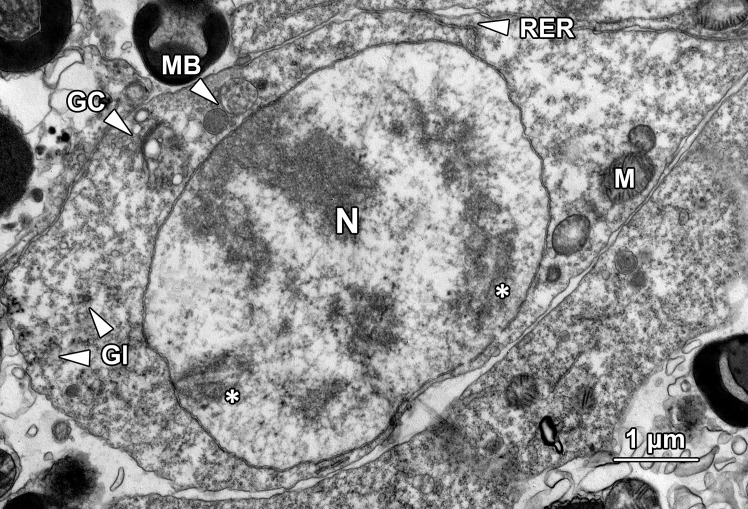
A transmission electron micrograph showing a primary spermatocyte at late zygotene/early pachytene stage of the first meiotic division. The most distinctive feature of the primary spermatocyte at this stage was the presence of synaptonemal complexes (*) in the nucleus. A Golgi complex with secreted irregular-sized vesicles and multivesicular bodies (MB) could be observed. Mitochondria (M) and glycogen (Gl) were scattered throughout the cytoplasm. RER, rough endoplasmic reticulum.

Primary spermatocytes were the end products of the mitotic divisions that spermatogonia underwent and were layered on the top of the type B spermatogonia. Primary spermatocytes exhibited an ovoid to fusiform shape about 8.1 ± 0.21 μm in diameter and an oval nucleus about 5.99 ± 0.16 μm in diameter (Fig 11). The primary spermatocytes were significant larger than the size of type B spermatogonia (P < 0.05). As the formation of primary spermatocytes involved a dramatic growth in cell volume, the cytoplasm was much more abundant than their precursors. The ribosomes appeared to be sparsely distributed throughout the cytoplasm, along with a small number of glycogen particles (Gl; Fig 11). Late zygotene/early pachytene primary spermatocytes could be characterised by the presence of the synaptonemal complex, a structure composed of proteins that formed a central shaft and two lateral bars associated with homologous chromosomes. The chromatin which previously lined the inner membrane of nuclear envelope disappeared at this stage and the double-membrane structure of nuclear envelope became conspicuous. The remaining portion of the nucleus was occupied with sparsely distributed euchromatin. A small number of mitochondria (M) and a conspicuous Golgi complex (GC) with secreted single membrane-bound vesicles and multivesicular bodies (MB) could be observed in the perinuclear area (Fig 11).

The first meiotic division of a primary spermatocyte generated two secondary spermatocytes. However, secondary spermatocytes were seldom observed during our ultrastructural investigation, suggesting the second meiotic division was a quick process that only lasted for a short period of time.

#### Spermatids

The newly formed young spermatids (YSt) were frequently found in pairs in the lumen of SE and NC compartments (Fig 5). The spermatids remained in pairs during early spermiogenesis and separated into individual spermatozoa after shedding most of their cytoplasm in the final stages of spermiogenesis. Based on their light microscope appearance, spermatids were divided into two major groups: non-flagellated young spermatids and flagellated older spermatids.

**Fig 12:**
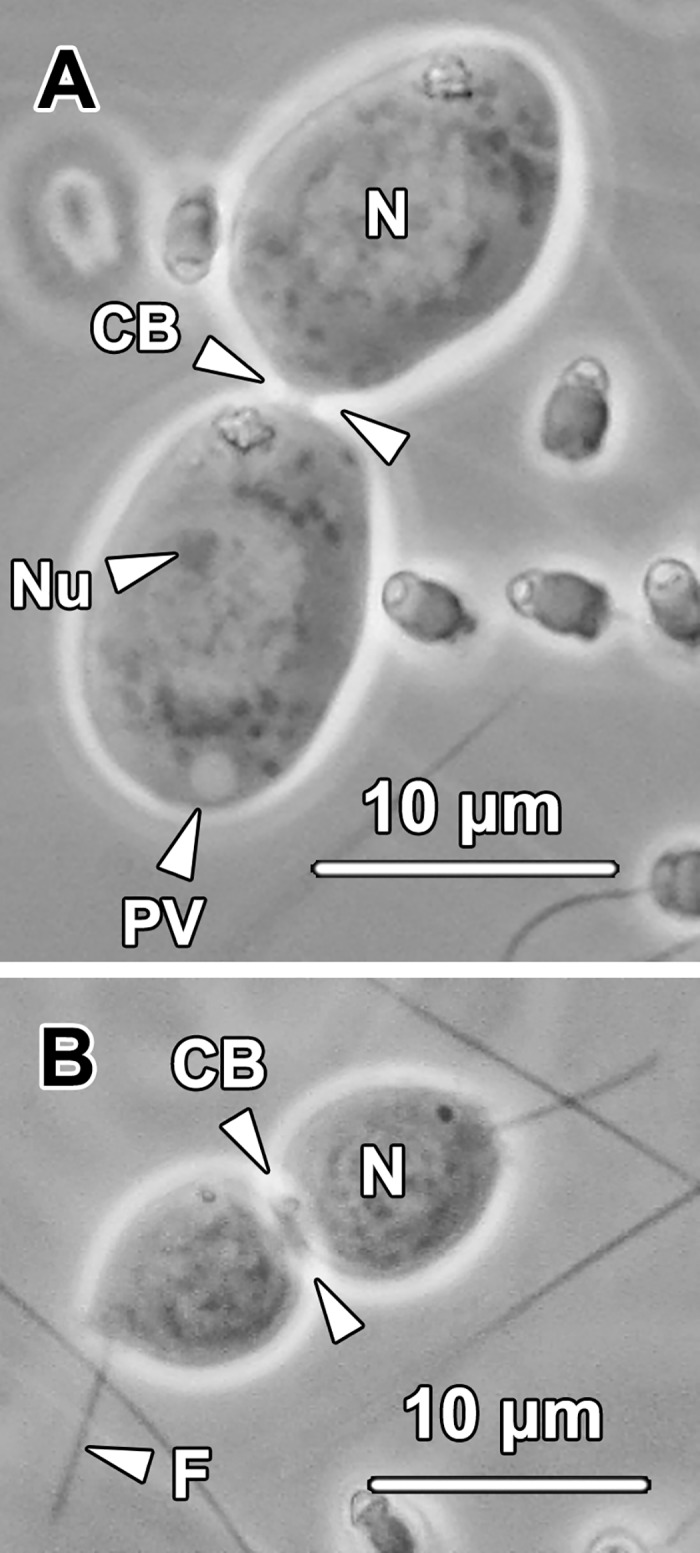
**(A) A light micrograph of a pair of young spermatids connected by a cytoplasmic bridge** (CB)**.** A proacrosomal vacuole (PV), which was the precursor of acrosome, could be seen adjacent to the oval- to spherical-shaped nucleus (N). A distinct nucleolus (Nu) was eccentrically situated in the nucleus. No flagellum had formed at this stage. **(B) A light micrograph of a pair of older spermatids connected by a cytoplasmic bridge** (CB)**.** At this stage, a forming sperm flagellum (F) was observed protruding from the posterior cytoplasm of the older spermatids. The older spermatids contained comparatively less cytoplasm and a spherical-shaped nucleus (N).

Non-flagellated young spermatids were found in pairs and connected by a common cytoplasmic bridge (CB; Fig 12A). They contained relatively abundant cytoplasm and were ovoid in shape with an oval- to spherical-shaped nucleus. A spherical proacrosomal vacuole (PV) could be observed in the posterior end of the cytoplasm opposite the cytoplasmic bridge. A distinct nucleolus (Nu) was frequently observed eccentrically located in the nucleus (N), or even attached to the nuclear membrane (Fig 12A).

Flagellated older spermatids remained in pairs (Fig 12B). Compared with the younger spermatids, the older ones were spherical in shape and contained less cytoplasm and a spherical nucleus (N) with denser chromatin. Notably, these round spermatids possessed an elongating flagellum (F) which protruded out of the cell body.

#### Spermatozoa

**Fig 13:**
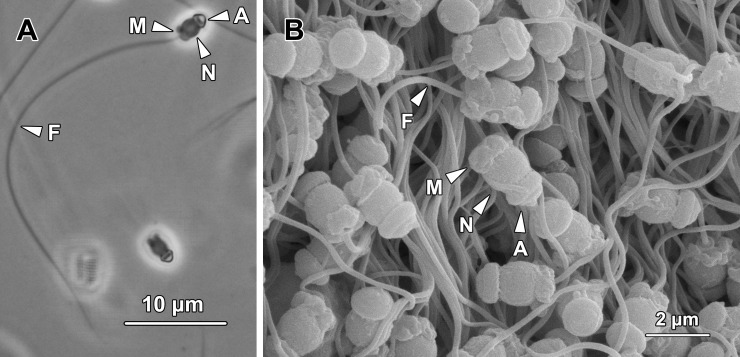
**(A) A light micrograph of a spermatozoon.** They consisted of a cap-like acrosome (A), an oval-shaped nucleus (N), a short midpiece (M) containing four round mitochondria and a fully elongated flagellum (F). **(B) A scanning electron micrograph showing bundles of spermatozoa stored in the lumen of the ED compartment.** A, acrosome; M, midpiece; N, nucleus; F, flagellum.

Spermatozoa in *G*. *caespitosa* could be observed mainly in the ED compartments of each germinal chamber (Fig 7). In both the efferent duct and vas deferens, the spermatozoa were mixed with a large number of spherical granules secreted by the nurse cells (Fig 5). Each spermatozoon consisted of a cap-like acrosome (A), an oval nucleus (N), a short midpiece (M) containing four round mitochondria, and an elongated flagellum (F; Fig 13). In live spermatozoa, the longitudinal length of the sperm head (including the acrosome, nucleus and midpiece) was approximately 3.35 μm and the length of flagellum was about 45 μm.

## Discussion

*Galeolaria caespitosa* is categorised into the annelid class Polychaeta, which comprises over 15,000 discovered species in 83 families [17, 18]. The reproductive characteristics and feeding strategies in polychaetes are extremely diverse, which may be attributed to the simplicity of their reproductive system and their remarkable plasticity and adaptability to a broad range of habitats [19, 20]. Although the reproductive biology of a considerable number of polychaetes has been documented previously, the structure of their male reproductive system has not been comprehensively described. The pattern of spermatogenesis, in particular the formation and differentiation of spermatogonia, has been neglected by a majority of previous studies. This is possibly because the identification of spermatogenic stages is relatively difficult in polychaetes given the ability of developing germ cells to detach from the seminiferous epithelium and are mixed with other cells at various stages of differentiation in the coelomic cavity.

In the present article, the pattern of spermatogenesis within the male reproductive tract of *G*. *caespitosa*, has been described for the first time. In *G*. *caespitosa*, the spermatogonia originated in the seminiferous epithelium lining the basement membrane of the germinal centre and were liberated into the lumen after the second meiotic division to complete the entirety of spermiogenesis while floating freely in the lumen. The germinal epithelium in *G*. *caespitosa* appeared as densely packed cell layers, where the type A and B spermatogonia occupied a position in close apposition to the basement membrane while primary and secondary spermatocytes were situated in the upper layers close to the lumen. The spermatogonia were tightly packed together, whereas the spermatocytes gradually lost intercellular adhesion generating cell layers with a less compacted appearance.

In other studied polychaetes, the structure and function of their testes are diverse but can be generally categorised into three distinct groups: complex, simple and non-definitive. Only a small number of polychaetes possess complex testes (e.g. *Branchipolynoe cf*. *seepensis* [21], *Petitia amphophthalma* [22]), where developing sperm cells are retained until the completion of spermiogenesis. These relatively complex testes are believed to facilitate sperm storage [21] and tend to be associated with internal fertilisation; their spermatozoa being transported into the female reproductive tract with the help of spermathecae [23].

By contrast, a large number of polychaetes possess simple testes surrounded by a thin layer of sheath/peritoneal cells or a simple layer of germinal epithelium, such as *Amathys lutzi* [24], *Marenzelleria viridis* [25], *Parergodrilus heideri* [26], *Phragmatopoma lapidosa* [27], *Pomatoceros triqueter* and *Pomatoceros lamarckii* [28], *Polydora* spp. and *Steblospio benedicti* [29] and *Stygocapitella subterranea* [30]. In addition, polychaetes with non-definitive testes, where the spermatogonia develop in cell clusters and are not invested by epithelial or sheath cells, are also very common, such as in *Capitella* spp., *Capitomastus* spp. and *Capitellides* spp. [31], *Gorgoniapolynoe caeciliae* [32], *Platynereis massiliensis* [33], *Sabella spallanzanii* [34, 35], *Vanadis formosa* and *Krohnia lepidota* [36]. In *G*. *caespitosa*, the spermatogonial stem cells arose from an unbound seminiferous epithelium and could therefore be categorised as a non-definitive testis. These studies on polychaetes with simpler testes indicate that the general pattern of their spermatogenesis is quite similar: spermatogenesis initially occurring in the testes after which spermatogenic cells are released into the coelomic cavity to complete their differentiation in relative isolation [37].

The spermatozoa of polychaetes have been categorised into three major types based on their function and morphology: ect-aquasperm, which are released into the water column where they fertilise similarly released oocytes; ent-aquasperm, which are also shed into the ambient water but are subsequently drawn into the female; and ect-aquasperm, which are completely isolated from seawater when being transferred from male to female [38]. Clearly, the types of spermatozoa in polychaetes are strongly associated with the fertilisation mechanisms that they adopt [39]–while ect-aquasperm are related to external fertilisation, ent-aquasperm and introsperm are correlated with sperm transfer, sperm storage in females and internal fertilisation. The spermatozoa in *G*. *caespitosa* have been classified as ect-aquasperm, based on its external fertilisation mode and primitive morphology of spermatozoa–a short sperm head containing a cap-like acrosome, an oval nucleus and a short midpiece [38].

Most of the polychaetes with complex testes, such as *Branchipolynoe cf*. *seepensis* [21] and *Petitia amphophthalma* [22], produce introsperm and adopt internal fertilisation. In comparison, the ones with simple or non-definitive testes produce varied types of spermatozoa and exhibit diverse fertilisation strategies. While a large number of polychaetes with simpler testes produce ect-aquasperm (e.g. *Amathys lutzi* [24], *Marenzelleria viridis*, *Phragmatopoma lapidosa*, *Sabella spallanzanii*), a considerable portion of them produce introsperm (e.g. *Parergodrilus heideri* [26], *Polydora* spp. and *Steblospio benedicti* [29], and *Stygocapitella subterranea* [30]). A small number of polychaetes produce ent-aquasperm, such as *Micromaldane* spp. [40], *Spirorbis morchi* [41], *Spirorbis spirorbis* [42] and Tomopteris helgolandica [43]; however, the structure of their testes has not been studied at present. Therefore, in light of the given information, the complex testes in polychaetes could be possibly related to the production of introsperm and the adoption of internal fertilisation strategy. However, polychaetes with simple or non-definitive testes have no consistency in their sperm morphology, sperm transfer mode or fertilisation strategy, suggesting that the reproductive characteristics of these species are not necessarily related to the simple structure of their testes.

In the abdominal region of *G*. *caespitosa*, blood vessels were found embedded in the intersegmental septa and situated near the seminiferous epithelium, but there was no physical contact between the blood vessels and any type of sperm cells. In contrast, in most studied polychaetes, the seminiferous epithelia, or even the entire testes have been found in intimate association with blood vessels such as the nephridial blood vessel (e.g. *Amathys lutzi* [24] and *Marenzelleria viridis* [25]), peritoneal blood vessel (e.g. *Phragmatopoma lapidosa* [27]), genital blood vessel (e.g. *Hesiocaeca methanicola* [17]) and parapodial blood vessel (e.g. *Steblospio benedicti* [29]). In most cases, spermatogonial stem cells are the only sperm cell type that have been observed directly attaching to the blood vessel, i.e. spermatogonia migrate peripherally during mitotic divisions and lose the direct connection with the circulation. However, in some polychaete species, such as *Gorgoniapolynoe caeciliae* [32], the sperm cells remain attached to the blood vessels until early spermiogenesis. In *Capitella* spp., clusters of blood cells have been observed near the testes in the coelomic cavity [31]. It is presumed that these ramified blood vessels nourish the early spermatogenic cells through intimate contact.

Although polychaetes with simpler testes display a similar pattern of spermatogenesis, the timing of germ cell detachment from the seminiferous epithelium is different. A number of previous studies discovered that germ cells were released from the epithelium during the final mitotic division or before the onset of the first meiotic division (e.g. *Amathys lutzi* [24], *Marenzelleria viridis* [25], *Phragmatopoma lapidosa* [27] and *Steblospio benedicti* [29]). The sperm cells in these species are released into the coelomic cavity early as spermatogonia. Noticeably, either the spermatogonia (e.g. *Amathys lutzi* [24]) or the spermatocytes (e.g. *Marenzelleria viridis* [25], *Phragmatopoma lapidosa* [27] and *Steblospio benedicti* [29]) in such species are in pairs and connected by a cytophore or cytoplasmic bridge. Distinct from these observations, sperm detachment from the seminiferous epithelium in *G*. *caespitosa* happened after the completion of second meiotic division. Moreover, no cytoplasmic bridge was observed between spermatogonia or spermatocytes in this species. In *Hydroides dianthus*, *Serpula vermicularis* and *Vermiliopsis infundibulum*, which are also serpulid polychaetes exhibiting similar reproductive characteristics (e.g. fertilisation strategy and sperm structure) with *G*. *caespitosa*, sperm detachment may happen after the completion of mitotic divisions due to the absence of spermatogonia in the coelomic cavity and similarly, no cytoplasmic bridge has been detected between spermatocytes [44]. Either way, these studies demonstrate that a significant portion of the spermatogenic process, occurs in the testicular lumen/coelomic cavity in these polychaete species.

In the germinal chambers of *G*. *caespitosa*, the whole ceiling of the seminiferous epithelium compartment was covered by a layer of ciliated epithelial cells, which facilitated the transportation of spermatids towards the nurse cell compartment. Ciliated intersegmental septa have also been observed in *Capitella* spp. [31], *Parergodrilus heideri* [26] and *Phragmatopoma lapidosa* [27]. The ciliated cells in these species, including the ones in *G*. *caespitosa*, exhibit a cubic to columnar shape and contain a spherical nucleus situated at the basal cytoplasm. Multiple cilia protrude from the anterior surface of the ciliated cells, whereas the basal surface of the cells firmly adheres to the intersegmental septum. Such ciliated epithelial cells have seldom been reported in other polychaete species. Nevertheless, they have been neglected or mistakenly recognised as sperm flagella in some polychaetes, as the histological sections presented in association with these studies do demonstrate the existence of ciliary structures (e.g. *Pomatoceros lamarckii* [28] and *Serpula vermicularis* [44]).

In *G*. *caespitosa*, each secondary spermatocyte divided into a pair of spermatids connected by a cytoplasmic bridge during the second meiotic division. The paired spermatids then underwent spermiogenesis simultaneously (Fig 12A and 12B). In contrast, in some polychaetes, a pair of secondary spermatocytes connected by a common cytoplasmic bridge develop into a tetrad of spermatids rather than two individual pairs of spermatids, such as in *Asetocalamyzas laonicola* [45], *Marenzelleria viridis* [25], *Phragmatopoma lapidosa* [27] and *Steblospio benedicti* [29]. Additionally, in a considerable number of other polychaetes, such as *Amathys lutzi* [24], *Boccardiella hamata* [46], *Capitella* spp. [31] and *Methanoaricia dendrobranchiata* [47], a large number of spermatocytes have been observed held together by a cytophore which tends to persist during the following spermiogenesis.

Apart from the support provided by blood vessels, no other type of supporting cell for sperm differentiation has been reported in any other polychaete species so far. Remarkably, nurse cells have been found in this study, occupying a considerable part of each germinal chamber in *G*. *caespitosa*. The entire wall of the two NC compartments was covered by these supporting cells, which contained numerous electron-dense granules (Fig 6). The nurse cells indirectly supported spermiogenesis by releasing their cellular contents into the lumen. According to *in vitro* culture studies we have conducted on the spermatids, the germinal fluid extracted from the abdominal chambers of *G*. *caespitosa* could support the differentiation of non-flagellated young spermatids into flagellated order spermatids (unpublished data). Therefore, the nurse cells were believed to play an important role in regulating the differentiation of spermatids in *G*. *caespitosa*. According to the scanning electron microscopic observation, the electron-dense secretory granules released by the nurse cells were not only found in the NC compartments, but also in the efferent duct (ED) compartments and vas deferens, suggesting that these granules might also be involved in maintaining the viability of spermatozoa during their transport and storage within the male reproductive tract.

During the entire process of spermatogenesis in *G*. *caespitosa*, developing germ cells have never been observed in direct association with any type of supporting cells. The haploid germ cells in *G*. *caespitosa*, in particular, were found floating independently in the lumen of the germinal chamber. In contrast, spermatogenesis in vertebrates is completely dependent on intimate contact with Sertoli cells [48]. This relative independence from supporting cells in *G*. *caespitosa* endowed this species great potential to be a model species for uncovering the molecular mechanisms underpinning sperm differentiation, particularly the complex process of spermatid differentiation, using *in vitro* cell culture techniques.

In summary, the germinal chamber of *G*. *caespitosa* appeared as a C-shaped tubule surrounding the main artery and intestine that run the whole length of the abdomen. A similar male reproductive system can be observed in histological sections of abdominal segments in *Aonides oxycephala* [49], *Branchiomma luctuosum* [50] and *Sabella spallanzanii* [34]. Based on morphological and functional differences, the germinal chamber in *G*. *caespitosa* was divided into three distinct compartments, including an SE compartment, two NC compartments and two ED compartments. The two ED compartments situated at the two ends of the C-shaped germinal chamber joined together to the vas deferens. Interestingly, this arrangement of compartments in the germinal chamber of *G*. *caespitosa* resembled the arrangement of the seminiferous tubule in mammalian testes. Thus the U-shaped seminiferous tubule in the human also has two openings running side by side to join the rete testis, efferent duct, and then the vas deferens [51]. This simple sessile annelid might therefore not only serve as a model bio-indicator species capable of revealing the presence of reproductive toxicants in the marine environment but also be used to shed light on such fundamental processes as spermiogenesis that could, in turn, inform our understanding of male reproduction across a wide range of species, including man.

## References

[1] Edgar GJ. Australian marine life: the plants and animals of temperate waters. Sydney: New Holland; 2008. p. 193.

[2] Styan CA, Kupriyanova EK, Havenhand JN. Barriers to cross‐fertilization between populations of a widely dispersed polychaete species are unlikely to have arisen through gametic compatibility arms‐races. Evolution. 2008;62(12): 3041–3055. 10.1111/j.1558-5646.2008.00521.x 18803690

[3] Dean HK. The use of polychaetes (Annelida) as indicator species of marine pollution: a review. Rev Biol Trop. 2008;56(4): 11–38.

[4] Melville F, Pulkownik A. Investigation of mangrove macroalgae as bioindicators of estuarine contamination. Mar Pollut Bull. 2006;52(10): 1260–1269. 10.1016/j.marpolbul.2006.02.021 16620872

[5] Halt MN, Kupriyanova EK, Cooper SJ, Rouse GW. Naming species with no morphological indicators: species status of *Galeolaria caespitosa* (Annelida: Serpulidae) inferred from nuclear and mitochondrial gene sequences and morphology. Invertebr Syst. 2009;23(3): 205–222.

[6] Bremner J. Species' traits and ecological functioning in marine conservation and management. J Exp Mar Biol Ecol. 2008;366(1–2): 37–47.

[7] Waring JS, Maher WA, Krikowa F. Trace metal bioaccumulation in eight common coastal Australian polychaeta. J Environ Monit. 2006;8(11): 1149–1157. 10.1039/b612509n 17075622

[8] Andrews J, Anderson D. The development of the polychaete *Galeolaria caespitosa* Lamarck (Fam. Serpulidae). Proc Linn Soc N S W. 1962;87(2): 185–188.

[9] Kupriyanova EK, Havenhand JN. Effects of temperature on sperm swimming behaviour, respiration and fertilization success in the serpulid polychaete, *Galeolaria caespitosa* (Annelida: Serpulidae). Invertebr Reprod Dev. 2005;48(1–3): 7–17.

[10] Marsden J, Anderson D. Larval development and metamorphosis of the serpulid polychaete *Galeolaria caespitosa* Lamarck. Mar Freshwater Res. 1981;32(4): 667–680.

[11] Ross K, Bidwell J. A 48-h larval development toxicity test using the marine polychaete *Galeolaria caespitosa* Lamarck (Fam. Serpulidae). Arch Environ Contam Toxicol. 2001;40(4): 489–496. 11525491 10.1007/s002440010201

[12] Hollows CF, Johnston EL, Marshall DJ. Copper reduces fertilisation success and exacerbates Allee effects in the field. Mar Ecol Prog Ser. 2007;333: 51–60.

[13] Kennedy AD, Jacoby CA. Biological indicators of marine environmental health: meiofauna–a neglected benthic component? Environ Monit Assess. 1999;54(1): 47–68.

[14] Bolton TF, Havenhand JN. Physiological versus viscosity-induced effects of water temperature on the swimming and sinking velocity of larvae of the serpulid polychaete *Galeolaria caespitosa*. Mar Ecol Prog Ser. 1997;159: 209–218.

[15] Falkenberg LJ, Havenhand JN, Styan CA. Sperm Accumulated Against Surface: A novel alternative bioassay for environmental monitoring. Mar Environ Res. 2016;114: 51–57. 10.1016/j.marenvres.2015.12.005 26763685

[16] Schlegel P, Havenhand JN, Obadia N, Williamson JE. Sperm swimming in the polychaete *Galeolaria caespitosa* shows substantial inter-individual variability in response to future ocean acidification. Mar Pollut Bull. 2014;78(1–2): 213–217. 10.1016/j.marpolbul.2013.10.040 24239098

[17] Eckelbarger K, Young C, Llodra ER, Brooke S, Tyler P. Gametogenesis, spawning behavior, and early development in the “iceworm” *Hesiocaeca methanicola* (Polychaeta: Hesionidae) from methane hydrates in the Gulf of Mexico. Mar Biol. 2001;138(4): 761–775.

[18] Hutchings P. Biodiversity and functioning of polychaetes in benthic sediments. Biodivers Conserv. 1998;7(9): 1133–1145.

[19] Wilson WH. Sexual reproductive modes in polychaetes: classification and diversity. Bull Mar Sci. 1991;48(2): 500–516.

[20] Giangrande A. Polychaete reproductive patterns, life cycles and life histories: an overview In: Ansell A, Gibson R, Barnes M, editors. Oceanography and marine biology: an annual review. 35 London: UCL Press; 1997 pp. 323–386.

[21] Van Dover CL, Trask J, Gross J, Knowlton A, Van Dover C. Reproductive biology of free-living and commensal polynoid polychaetes at the Lucky Strike hydrothermal vent field (Mid-Atlantic Ridge). Mar Ecol Prog Ser. 1999;181: 201–214.

[22] Bührmann C, Westheide W, Purschke G. Spermatogenesis and sperm ultrastructure in the interstitial syllid *Petitia amphophthalma* (Annelida, Polychaeta). Ophelia. 1996;45(2): 81–100.

[23] Hilário A, Young CM, Tyler PA. Sperm storage, internal fertilization, and embryonic dispersal in vent and seep tubeworms (Polychaeta: Siboglinidae: Vestimentifera). Biol Bull. 2005;208(1): 20–28. 10.2307/3593097 15713809

[24] Blake EA, Van Dover CL. The reproductive biology of *Amathys lutzi*, an ampharetid polychaete from hydrothermal vents on the Mid-Atlantic Ridge. Invertebr Biol. 2005;124(3): 254–264.

[25] Bochert R. An electron microscopic study of spermatogenesis in *Marenzelleria viridis* (Verrill, 1873) (Polychaeta: Spionidae). Acta Zool. 1996;77(3): 191–199.

[26] Purschke G. Male genital organs, spermatogenesis and spermatozoa in the enigmatic terrestrial polychaete *Parergodrilus heideri* (Annelida, Parergodrilidae). Zoomorphology. 2002;121(3): 125–138.

[27] Eckelbarger KJ. Ultrastructure of spermatogenesis in the reef-building polychaete *Phragmatopoma lapidosa* (Sabellariidae) with special reference to acrosome morphogenesis. J Ultrastruct Res. 1984;89(2): 146–164.

[28] Cotter E, O'Riordan R, Myers A. A histological study of reproduction in the serpulids *Pomatoceros triqueter* and *Pomatoceros lamarckii* (Annelida: Polychaeta). Mar Biol. 2003;142(5): 905–914.

[29] Rice SA. Spermatogenesis and sperm ultrastructure in three species of *Polydora* and in *Streblospio benedicti* (Polychaeta: Spionidae). Zoomorphology. 1981;97(1–2): 1–16.

[30] Purschke G, Fursman MC. Spermatogenesis and spermatozoa in *Stygocapitella subterranea* (Annelida, Parergodrilidae), an enigmatic supralittoral polychaete. Zoomorphology. 2005;124(3): 137–148.

[31] Eckelbarger K, Grassle J. Spermatogenesis, sperm storage and comparative sperm morphology in nine species of *Capitella*, *Capitomastus* and *Capitellides* (Polychaeta: Capitellidae). Mar Biol. 1987;95(3): 415–429.

[32] Eckelbarger K, Watling L, Fournier H. Reproductive biology of the deep-sea polychaete *Gorgoniapolynoe caeciliae* (Polynoidae), a commensal species associated with octocorals. J Mar Biol Assoc U K. 2005;85(06): 1425–1433.

[33] Lücht J, Pfannenstiel H-D. Spermatogenesis in *Platynereis massiliensis* (Polychaeta: Nereidae). Helgolander Meeresunters. 1989;43(1): 19–28.

[34] Giangrande A, Licciano M, Pagliara P, Gambi M. Gametogenesis and larval development in *Sabella spallanzanii* (Polychaeta: Sabellidae) from the Mediterranean Sea. Mar Biol. 2000;136(5): 847–861.

[35] Currie DR, McArthur MA, Cohen BF. Reproduction and distribution of the invasive European fanworm *Sabella spallanzanii* (Polychaeta: Sabellidae) in Port Phillip Bay, Victoria, Australia. Mar Biol. 2000;136(4): 645–656.

[36] Rice SA, Eckelbarger KJ. An ultrastructural investigation of spermatogenesis in the holopelagic polychaetes *Vanadis formosa* and *Krohnia lepidota* (Polychaeta: Alciopidae). Biol Bull. 1989;176(2): 123–134.29300594

[37] Rouse GW. The Annelida In: Anderson DT, editor. Invertebrate zoology. South Melbourne: Oxford University Press; 1998 pp. 196–200.

[38] Jamieson BGM, Rouse GW. The spermatozoa of the Polychaeta (Annelida): An ultrastructural review. Biol Rev. 1989;64(2): 93–157. 2675996 10.1111/j.1469-185x.1989.tb00673.x

[39] Rouse GW. Polychaete sperm: phylogenetic and functional considerations. Hydrobiologia. 1999;402(0): 215–224.

[40] Rouse GW. Ultrastructure of sperm and spermathecae in *Micromaldane* spp. (Polychaeta: Capitellida: Maldanidae). Mar Biol. 1992;113(4): 655–668.

[41] Daly JM, Golding DW. A description of the spermatheca of *Spirorbis spirorbis* (L.) (Polychaeta: Serpulidae) and evidence for a novel mode of sperm transmission. J Mar Biol Assoc UK. 1977;57(1): 219–227.

[42] Picard A. Spermatogenesis and sperm—spermatheca relations in Spirorbis spirorbis (L.). Int J Invertebr Reprod. 1980;2(2): 73–83.

[43] Franzén Å. Ultrastructure of the biflagellate spermatozoon of *Tomopteris helgolandica* Greef, 1879 (Annelida, Polychaeta). Gamete Res. 1982;6(1): 29–37.

[44] Gherardi M, Lepore E, Sciscioli M, Mercurio M, Licciano M, Giangrande A. A study on spermatogenesis of three Mediterranean serpulid species. Ital J Zool. 2011;78(2): 174–181.

[45] Vortsepneva EV, Zhadan AE, Tzetlin AB. Spermiogenesis and sperm ultrastructure of *Asetocalamyzas laonicola* Tzetlin, 1985 (Polychaeta), an ectoparasite of the large spionid *Scolelepis* cf. *matsugae* Sikorsfi, 1994, from the White Sea. Sci Mar. 2006;70(S3): 343–350.

[46] Reunov AA, Yurchenko OV, Alexandrova YN, Radashevsky VI. Spermatogenesis in *Boccardiella hamata* (Polychaeta: Spionidae) from the Sea of Japan: sperm formation mechanisms as characteristics for future taxonomic revision. Acta Zool. 2010;91(4): 447–456.

[47] Eckelbarger KJ, Young CM. Spermiogenesis and modified sperm morphology in the “seepworm” *Methanoaricia dendrobranchiata* (Polychaeta: Orbiniidae) from a methane seep environment in the Gulf of Mexico: implications for fertilization biology. Biol Bull. 2002;203(2): 134–143. 10.2307/1543382 12414563

[48] Jones R, Lin M. Spermatogenesis in birds. Oxf Rev Reprod Biol. 1992;15: 233–264. 8336978

[49] Radashevsky VI, Alexandrova YN, Yurchenko OV. Spermiogenesis and spermatozoa ultrastructure of *Aonides oxycephala* (Annelida: Spionidae) from the Sea of Japan. Invertebr Reprod Dev. 2011;55(3): 168–174.

[50] Licciano M, Giangrande A, Gambi MC. Reproduction and simultaneous hermaphroditism in *Branchiomma luctuosum* (Polychaeta, Sabellidae) from the Mediterranean Sea. Invertebr Biol. 2002;121(1): 55–65.

[51] Bloom W, Fawcell D. A textbook of histology. Philadelphia: W. B. Saunders; 1975 pp. 805–855.

